# Effects of Orthotic Interventions on Pain, Balance, and Plantar Pressure in Flexible Flatfoot: A Systematic Review

**DOI:** 10.1002/jfa2.70218

**Published:** 2026-09-30

**Authors:** Taeseok Choi, Sangbin Lee, Wansuk Choi, Seoyoon Heo

**Affiliations:** ^1^ Department of Physical Therapy Kunjang University College Gunsan‐Si South Korea; ^2^ Department of Physical Therapy Namseoul University Cheonan‐Si South Korea; ^3^ Department of Physical Therapy Kyungwoon University Gumi‐Si South Korea; ^4^ Department of Occupational Therapy Kyungbok University Namyangju‐Si South Korea

**Keywords:** biomechanics, foot orthoses, pain management, pes planus, postural balance

## Abstract

**Objective:**

To systematically review the evidence for orthotic interventions on pain, balance, and plantar pressure distribution in individuals with flexible flatfoot.

**Methods:**

Randomized and nonrandomized controlled trials of orthotic interventions for flexible flatfoot were included. Primary outcomes were pain, balance, and plantar pressure. MEDLINE via PubMed, EMBASE via Elsevier, and the Cochrane Library were searched from inception to October 16, 2025. All effect sizes were independently recomputed, and pooling required two or more compatible datasets.

**Results:**

Eight studies with 382 participants were included: six randomized controlled trials, one nonrandomized controlled trial, and one within‐subject repeated‐measures study. No domain met the pooling condition, so all previously reported summary estimates and heterogeneity statistics are withdrawn. Pain against a comparator without arch support rested on Yurt et al. (*n* = 67): *g* = −1.02 [95% CI: −1.54, −0.49] on the trial's designated primary outcome, a 100 mm visual analog scale, but *g* = −0.28 on its Foot Function Index total. In Elsayed et al. (*n* = 40) both arms wore the same orthosis, so *g* = −0.97 [−1.62, −0.33] estimates added exercise, not the device. Balance rested on Yalfani et al. (*n* = 30): *g* = −0.86 [−1.59, −0.13]. In Khodaei et al. (*n* = 19), plantar load was redistributed rather than reduced, moving from the heel and lesser metatarsals (*g* = −1.93 to −1.09) to the medial midfoot (+1.76). Taspinar et al. were withdrawn as arithmetically irreproducible.

**Conclusions:**

Every domain rests on a single small study, so no general efficacy claim is supportable. The most defensible finding is mechanical: load shifts from the heel and lesser metatarsals to the medial midfoot, as a medial arch support is designed to do. The pain estimate is not robust to instrument choice within its own trial. GRADE certainty is very low for every outcome; certainty for plantar pressure began at low because the contributing study did not randomize allocation. The two nonrandomized studies were additionally appraised with ROBINS‐I, at critical and serious risk, respectively. Adequately powered randomized trials with a defined comparator and a pre‐specified primary outcome are required.

## 1 Introduction

Flexible flatfoot is characterized by the partial or complete collapse of the medial longitudinal arch during weight‐bearing activities, often accompanied by hindfoot valgus and forefoot abduction [1, 2]. This condition is common across the lifespan, affecting both children and adults, and is associated with musculoskeletal complaints including lower extremity pain, joint dysfunction, and altered gait patterns [3, 4, 5]. The biomechanical alterations associated with flexible flatfoot lead to abnormal load distribution across the foot, contributing to compensatory mechanisms that affect overall posture and movement [6].

Foot orthoses and insoles are the mainstay of conservative treatment for flexible flatfoot. Current evidence and structured nonoperative protocols [7], together with systematic reviews of conservative management [8], endorse orthotic devices to support the medial longitudinal arch and reduce symptoms. Their intended mechanism is to redistribute plantar pressure during weight‐bearing. Studies by Oerlemans et al. [9] and Huang et al. [10] have demonstrated the potential effectiveness of orthotic interventions in reducing pain and enhancing functional outcomes. However, the heterogeneity in outcome measures and methodological approaches has led to varying conclusions about their therapeutic value.

Pain is a key outcome because it directly affects patients' daily functioning and quality of life [11]. Individuals with flexible flat feet often report various pain patterns, including arch pain, heel pain, and general foot fatigue. Balance is a second relevant outcome, because the altered foot posture can reduce proprioceptive input from the plantar surface and impair postural control [12, 13]. Studies have shown that individual with flexible flatfoot exhibit greater postural sway and compromised stability compared with those with normal foot arches [14, 15]. Plantar pressure measurement provides an objective way to quantify how flatfoot and its treatment alter foot loading.

Despite the widespread use of orthotic interventions for flexible flatfoot, there has been no comprehensive synthesis of evidence examining their effects across multiple outcome domains. While previous reviews have focused on specific aspects of orthotic intervention or particular outcome measures, no review has pooled pain, balance, and plantar pressure outcomes for this condition in a single analysis. Assessing these outcomes together allows the effects of orthoses on symptoms and on foot mechanics to be compared within one review.

Therefore, this study aims to systematically review the effects of orthotic interventions on pain, balance, and plantar pressure distribution in individuals with flexible flatfoot. By synthesizing evidence across these three critical outcome domains, this review aims to inform the clinical management of flexible flatfoot and to identify gaps for further research.

## 2 Methods

### 2.1 Study Design

A systematic review was performed following PRISMA guidelines [16]. The protocol was prospectively registered in PROSPERO (CRD42025649490). No amendment to the registered protocol was filed while the review was being conducted; an amendment recording the deviations declared in this manuscript, including those introduced at revision, has since been filed with the PROSPERO registry; the narrowing of the plantar variable to peak pressure, declared below, will be added at the next update of that record. Three procedures specified in the protocol were not carried out as planned: screening of the reference lists of included reports, the pre‐specified subgroup and meta‐regression analyses, and the planned pooling of effect estimates within each outcome domain. Each deviation is described in the sections below. The search was run to October 16, 2025 rather than to the February 16, 2025 date named in the protocol; the protocol provided for the search to be updated before the final analysis, so this is an update rather than a departure. The appraisal of nonrandomized studies with ROBINS‐I was specified in the protocol and is not a deviation either. The eligibility criteria are otherwise those of the protocol, with the design descriptions in criteria (1) and (4) made explicit at revision to name a within‐subject design that was included but not named in the protocol. This study examined the biomechanical and clinical impacts of orthotic interventions on the flexible flatfoot, focusing on pain, balance, and plantar pressure distribution.

### 2.2 Literature Search and Study Selection

A comprehensive literature search was conducted using MEDLINE via PubMed, EMBASE via Elsevier, and Cochrane Library from inception until October 16, 2025. The search strategy combined Medical Subject Headings (MeSH) terms and text words: “Foot Orthoses” (including “Orthotic Devices”, “Foot Arch Supports”, “Orthotic Shoe Inserts”) AND “Flatfoot” (including “Pes Planus”, “Flexible Flatfoot”). Similar terms were adapted for EMBASE (EMTREE terms) and Cochrane Library. The complete search yielded 178 articles from PubMed, 40 from EMBASE, and 72 from the Cochrane Library. The full electronic search strings for each database, including all MeSH and EMTREE terms, field tags, Boolean operators, and applied limits, together with the date the search was run and the number of records retrieved from each source, are reported in Supporting Information S1.

After removing 45 duplicates, two independent reviewers (TS, SB) screened 245 records by title and abstract. A full‐text review of 59 potentially eligible articles was conducted by another pair of reviewers (SY, WS). Disagreements were resolved through discussion with a third reviewer (TS). Eight articles met all inclusion criteria for final analysis (Figure 1). Reviewers were not blinded to study authors, institutions, or journals during screening; screening was performed independently and in duplicate to mitigate selection bias.

**FIGURE 1:**
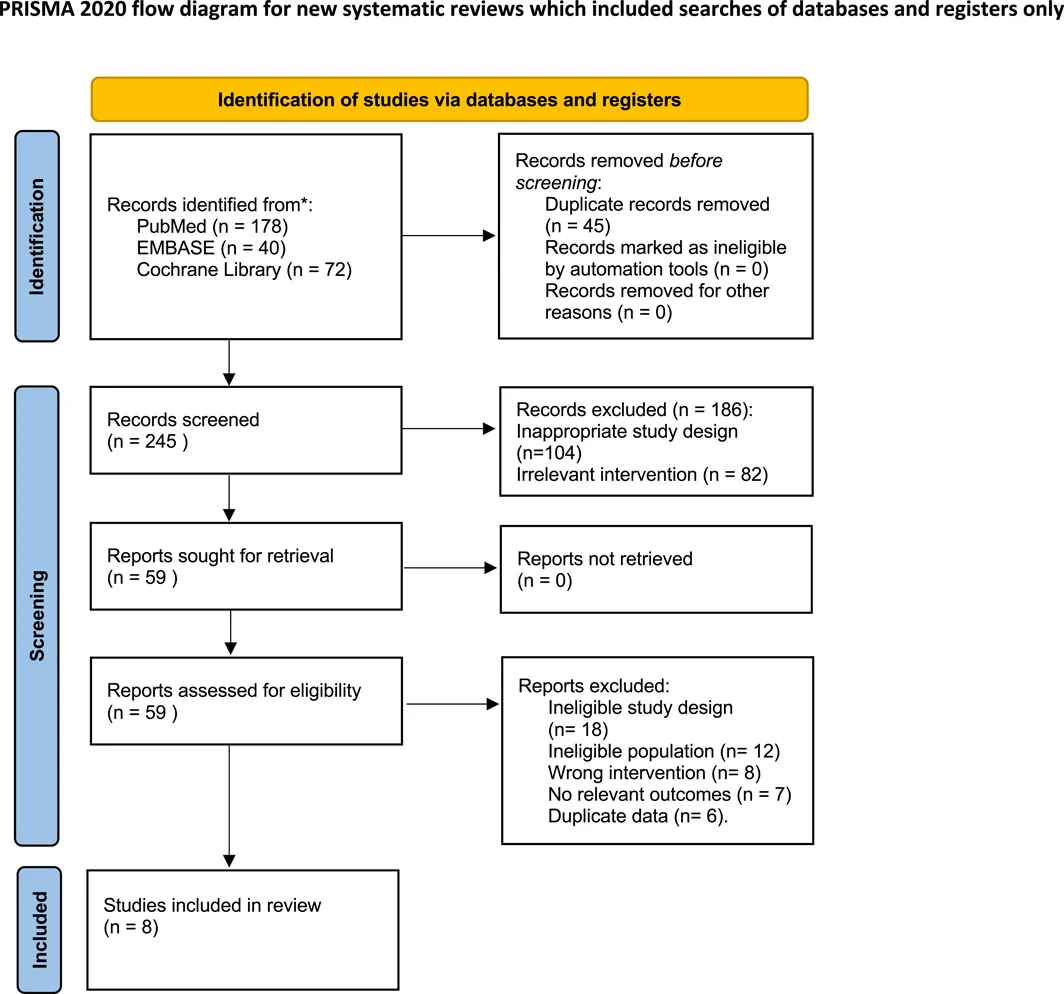
PRISMA flow diagram.

### 2.3 Language and Source Restrictions

As specified in the registered protocol, eligibility was restricted to reports available in full text in English; non‐English reports were not translated, and a language bias therefore cannot be excluded. Retrieval was limited to the three electronic databases named above. Clinical‐trial registries, including ClinicalTrials.gov and the WHO International Clinical Trials Registry Platform, were not searched, and no gray literature, such as conference abstracts, dissertations, or unpublished reports, was sought. Reference lists of the included articles and of relevant reviews were not screened, and no forward citation tracking was performed. Unpublished or non‐English trials meeting the eligibility criteria would consequently not have been identified, and the implications of this for publication bias are considered in the limitation section.

### 2.4 Inclusion and Exclusion Criteria

Studies were included if they met the following criteria: (1) randomized controlled trials, quasi‐randomized controlled trials, controlled clinical trials, or within‐subject repeated‐measures studies in which each participant served as his or her own control, evaluating orthotic interventions; (2) participants of any age diagnosed with flexible flatfoot by clinical assessment or radiographic imaging (e.g., Foot Posture Index, navicular drop test, or radiographic arch measures); (3) orthotic intervention, including prefabricated or custom‐made foot orthoses, insoles, or arch supports, administered alone or in combination with other conservative treatments; (4) control group or control condition receiving no intervention, a placebo or sham device, or a different orthotic intervention; (5) outcome measures including pain, balance or postural stability, or plantar pressure; (6) full text available in English.

Studies were excluded if participants had rigid flatfoot, neurological conditions affecting foot biomechanics, previous foot surgery, or if interventions were surgical or pharmacological, or consisted solely of non‐orthotic physical therapy without an orthotic component.

### 2.5 Study Designs and Their Designation

The eight included studies do not share one design, and the collective label “controlled trials” used in earlier versions of this manuscript did not describe all of them accurately. Six studies allocated participants at random. Five of these allocated participants to parallel groups [17, 18, 19, 20, 21]; in the sixth [22] each participant was measured under every condition and only the order of the conditions was randomized. One of the five [18] ran parallel groups to 6 months and then crossed participants over, so only its first period would have been eligible had it contributed an estimate. One study allocated participants to parallel groups by patient preference rather than at random [23]. One recruited by non‐random convenience sampling and compared orthotic conditions within the same participants, each participant serving as his or her own control [24]; that design is not a controlled trial in the parallel‐group sense at all. Neither is the condition‐order design of [22], although that study did randomize. We therefore no longer apply a single collective label to all eight. They are referred to collectively as the included studies, and the design of each is named wherever its design bears on the analysis, as a randomized controlled trial, a nonrandomized controlled trial, or a within‐subject repeated‐measures study. Retaining a label broad enough to cover all eight would have concealed precisely the design differences that determine which risk‐of‐bias instrument applies, whether a contrast is paired or unpaired, and how much weight the resulting estimate can carry.

### 2.6 Data Extraction and Quality Assessment

Data extraction was conducted by one pair of reviewers (TS, SB) using a standardized form, including study characteristics, participant demographics, intervention details, control group characteristics, outcome measures, and results. Extracted items also covered the type and construction of the orthosis, the prescribed wearing regimen, any co‐interventions delivered to the experimental or control arms, and the duration of follow‐up. A second pair of reviewers (SY, WS) independently verified data accuracy. Discrepancies were resolved through discussion. Arm‐level means, standard deviations, and sample sizes were recorded for every eligible outcome, instrument, and time point that a study reported, so that the choice among them was made by rule rather than at the moment of extraction; the rules applied are set out under Outcome Measures and Domain Extraction Rules below. Where a study supplied more than one intervention arm, or measured the same participants under more than one condition, the arms or conditions were not entered as separate independent datasets; the remedies applied instead are set out under Unit of Analysis below. The population, orthosis type, comparator, co‐interventions, outcome measures, and follow‐up of each included study are summarized in Supporting Information S2.

#### 2.6.1 Independent Re‐Extraction and Verification of Effect Estimates

Two errors in the pain estimates of an earlier version of this manuscript were identified during peer review. An extraction error of that kind arises from the selection of an instrument, a time point, or a pair of arms to contrast, and none of those selections is a property of the outcome domain in which the error occurs. The audit was therefore not confined to the domain in which the errors were found. Every effect estimate in the review was re‐extracted and recomputed from the primary reports, in the pain, balance, and plantar pressure domains alike, and no estimate was carried forward from the previous version. Two reviewers (TS, SY) worked independently and from the published reports only, without sight of the previously extracted values or of each other's figures, recording for each estimate the table or figure of origin, the instrument, the time point, the two arms or conditions contrasted, the direction of the scale, and whether the value was a final value or a change from baseline. Each reviewer computed Hedges' g and its standard error from the arm‐level means, standard deviations, and sample sizes [25]. The two sets of figures were then compared, and any difference beyond rounding was adjudicated by a third reviewer (SB) against the source report. An audit trail was compiled for every estimate in this review, giving for each the source table or figure, the instrument, the time point, the two arms or conditions contrasted, the arm‐level means, standard deviations and sample sizes, and the full computation of Hedges' g and its standard error. It covers the estimates that are reported and, equally, those that are not, with the ground for each exclusion, and it is available from the corresponding author on request.

Two checks were then applied uniformly to every estimate, whether or not it had been queried. First, each standard error was compared with the smallest standard error arithmetically attainable for the sample sizes of the contrast concerned, which for an independent‐group comparison is the value taken at a standardized mean difference of zero. A standard error below that floor cannot arise from the reported sample sizes under any analysis, and identifies an error in the estimate or in the sample sizes rather than an unusually precise result; the check requires no assumption about which analysis a study performed. Second, where an estimate could not be reproduced directly from the source report, we enumerated an exhaustive grid of the candidate computations that could have produced it, crossing every instrument the study reported, every time point, every pairwise contrast between arms, and both final‐value and change‐from‐baseline analyses at imputed pre‐post correlations from 0.0 to 0.9 in steps of 0.1, with the change score standardized alternatively by the baseline standard deviation. An estimate that no computation in that grid reproduced to within 0.005 was treated as unverifiable and withdrawn from the synthesis, with the grounds reported in the Results.

Risk of bias was assessed using the Cochrane Risk of Bias 2 (RoB 2) tool for five domains: randomization process, deviations from intended interventions, missing outcome data, measurement of the outcome, and selection of the reported result [26]. Each domain was classified as low risk, high risk, or some concerns by two independent reviewers (SY, WS). RoB 2 presupposes randomized allocation and does not assess confounding factors, so the two studies that did not randomize were additionally appraised with ROBINS‐I [27]. The registered protocol specified this instrument for nonrandomized studies, so its use here follows the protocol rather than departing from it [28]; the first ROBINS‐I domain addresses confounding and is decisive where allocation followed patient preference. ROBINS‐I judgments are reported as low, moderate, serious, or critical risk of bias. The two instruments are not interchangeable, and their ratings are reported separately rather than merged into a single ordering across the eight studies.

### 2.7 Outcome Measures and Domain Extraction Rules

Three main outcomes were assessed at standardized time points. Pain was evaluated using Visual Analog Scale (VAS) or Numeric Rating Scale (NRS), with minimal clinically important difference defined as ≥ 2 points on VAS. Balance was assessed using the BALANCE SYSTEM Biodex device for dynamic stability measures and Kistler force plates for center of pressure parameters, following standardized protocols. Plantar pressure distribution was analyzed using pressure platforms during standardized walking trials. The navicular drop test was conducted according to the Brody method (intraclass correlation coefficient [ICC] = 0.83–0.95).

Which of a study's reported values entered the synthesis was fixed by rule, and each rule below was applied to every study in the same way and before any effect estimate was inspected. Two conventions apply across all three domains. Final values at the designated endpoint were used in preference to change scores, because a change‐score analysis requires a pre‐post correlation that no included study reported and that must therefore be imputed; where both analyses were computable, they are compared in the sensitivity analyses described below. Where a study measured an outcome both with the orthosis in place and with it removed, the measurement condition was recorded as part of the outcome definition, because the two conditions answer different questions: a measurement taken with the device in situ estimates its immediate mechanical action, and a measurement taken with the device removed after a period of wear estimates an adaptation that persists without it.

For pain, the instrument that the study itself designated as its primary outcome was used, at the time point the study designated as its primary endpoint. Where a study designated no primary outcome, the direct pain‐intensity scale, a visual analog scale, or a numeric rating scale, was used in preference to the pain subscale or total score of a multidimensional function questionnaire, and the latest time point within the short‐term window was taken. A study whose pre‐specified pain instrument was not reported at all contributed no pain estimate, because selecting whichever pain‐related value the report did happen to contain would substitute our choice of outcome for the study's own. Pain contrasts were then classified by what the comparator provided, into stratum A, an orthosis against a comparator providing no arch support, whether a sham insole, a shoe alone, or no device; stratum B, one orthosis against a different orthosis; and stratum C, an orthosis with an added adjunct intervention against the same orthosis alone. The three strata estimate different quantities, and an estimate from one stratum was never combined with an estimate from another.

For balance and postural control, where a study reported a composite stability index together with its directional components, the composite index was used and the components were not entered separately, since the components are arithmetic constituents of the composite and entering both would represent the same participants more than once within one analysis. Where a study reported several distinct balance constructs, the construct closest to standing postural stability was used. Balance estimates were eligible to be combined only if they were obtained under the same measurement condition in the sense defined above; a stability index measured with the orthosis removed and a center‐of‐pressure parameter measured with the device in situ were not treated as measurements of one quantity.

For plantar loading, peak plantar pressure was taken as the loading variable, and every anatomical region a study reported it for was extracted and retained as a separate estimate for that study. Peak pressure expresses load per unit area at a region, which is the quantity in which redistribution is defined. Two other plantar variables appear in the included reports and were extracted but not used as loading estimates. Contact area, which the registered protocol also named, increases by construction when a device conforms to the medial arch, so it measures how closely the device fits rather than what load the region carries; therefore, a study reporting contact area alone contributes no loading estimate. The maximum force is not normalized to the area over which it acts and thus does not answer the redistribution question. Confining the plantar variable to peak pressure narrows the protocol's outcome list and is declared as a deviation. Regional values were not averaged into a single foot‐level figure, and no one region was nominated to represent the foot. Plantar load is redistributed rather than removed: a device that unloads one region necessarily loads another, so regions move in opposite directions by construction, and an average across them tends toward zero, however large the redistribution. A single summary figure would therefore report the mechanical action of an arch support as small at exactly the point where it is large. The regional estimates from a study are reported together as a redistribution profile, with each region carrying its own confidence interval, and are not pooled with one another.

### 2.8 Data Synthesis

Before any estimate was pooled, the datasets within each outcome domain were tested against three conditions for combinability, all of which had to hold. They had to estimate the same contrast, in the sense of falling within the same comparator stratum defined above. They had to be obtained on the same construct and under the same measurement conditions. They had to come from independent participants, or to have been reduced to one estimate per study by the remedies described under Unit of Analysis below. Only datasets satisfying all three conditions were eligible for pooling. Had two or more eligible datasets remained within a domain, they would have been pooled using Comprehensive Meta‐Analysis software version 3.0 (Biostat, Englewood, NJ) with a random‐effects model and the DerSimonian‐Laird estimator [29], effect sizes being expressed as standardized mean differences (Hedges' g) with 95% confidence intervals [25].

Applying these conditions left a single eligible dataset in every outcome domain, and no pooled estimate is reported anywhere in this review. In the pain domain, the eligible contrasts fell into two different comparator strata, leaving one estimate in each. In the balance domain, the two candidate studies measured under opposite conditions, one with the orthosis removed after a period of wear and one with the device in situ. In the plantar loading domain, the sole admissible study contributes a regional profile whose regions are not commensurable with one another. Where a domain holds one estimate, a random‐effects model is not estimable, between‐study heterogeneity is undefined rather than zero and the I^2^ statistic that would ordinarily quantify it [30] is not computable, and a summary diamond would depict a single study while carrying the visual authority of a synthesized body of evidence. All pooled estimates, all heterogeneity statistics, and all summary diamonds reported in earlier versions of this manuscript are therefore withdrawn. Each domain is reported instead as a single study estimate with its 95% confidence interval, or as a regional profile, together with a narrative account of the studies that were not combinable with it.

Three conventions from the registered protocol survived the withdrawal of the pooled analyses. Time points were classified as short‐term (up to 3 months), medium‐term (> 3 to 6 months), and long‐term (> 6 months). Conversion of medians and interquartile ranges to means and standard deviations was planned in accordance with Cochrane Handbook recommendations [28, 31], but proved unnecessary, as set out below. Publication bias assessment using funnel plots and Egger's test was planned for any domain to which more than 10 studies contributed, and no domain approached that number.

#### 2.8.1 Unit of Analysis

Four studies were represented more than once within a single analysis in the previous version of this review: Taspinar et al. [23] in the pain analysis, Saeedi et al. [22] in the balance analysis, and Xu et al. [19] and Elsayed et al. [20] in the plantar pressure analysis. One further study, Yurt et al. [21], had more than one eligible intervention arm available. Entering the same participants twice within one analysis is a unit‐of‐analysis violation: the observations are not independent, the study receives more weight than its sample size warrants, and the precision of the result is overstated. A remedy from the Cochrane Handbook [28] was applied to each study that supplied more than one eligible dataset, selected according to the reason the study supplied more than one. Where a study had two or more eligible intervention arms of different participants sharing a single control arm, the eligible arms were combined into one group and compared with the shared control once, using the Handbook formula for combining groups given in Section 6.5.2.10, which pools the arm means weighted by sample size and recovers the standard deviation of the combined group from its within‐arm and between‐arm components [28]. Splitting the shared control instead would have preserved two rows in the analysis at the cost of halving the control sample arbitrarily, and combining is preferred where the arms are variants of one intervention rather than distinct treatments. In Yurt et al. [21], the two active orthosis arms were combined in this way and compared once with the sham insole arm. In Saeedi et al. [22], the two orthosis conditions were measured on the same 34 participants rather than in separate arms, so the within‐participant rule described next would have applied had that study entered a synthesis; because it is reported narratively, its two conditions are described separately and neither contributes to an estimate to which any other study also contributes.

Where a study measured the same participants under more than one condition, the resulting datasets are paired rather than independent, and the Cochrane Handbook treatment of within‐participant designs applies [28]. In Khodaei et al. [24], each participant was measured under two orthotic conditions and under a shoe‐only condition. The two orthotic conditions were averaged within each participant to a single orthosis value, using the rule for combining correlated conditions measured on the same people rather than the independent‐subgroups formula applied to Yurt et al. [21]; the independent‐subgroups formula would return the standard deviation of a pooled set of 38 distinct participants, and no such set exists. The contrast against the shoe‐only condition was then computed within participants, so that the 19 participants entered each regional estimate exactly once. The standard error of a paired contrast depends on the correlation between the paired measurements, which the report does not provide; it was assumed to be 0.5, the conventional value in the absence of information, and was varied in the sensitivity analyses described below. Because the two orthosis conditions are combined before the contrast is taken, the assumed correlation enters the combined standard deviation and therefore the standardizer, so it moves the point estimate as well as its standard error. The movement is small and is quantified in the Results. The standardizer is the between‐participant standard deviation of the raw scores, not the standard deviation of the within‐participant differences, so that the estimate remains on the same scale as an independent‐group standardized mean difference. Where a single pair of conditions is contrasted, the assumed correlation would enter only the standard error; here it also enters the standardizer, because the intervention side is itself a combination of two correlated conditions. Regions within this study remain separate estimates and are not combined with one another, for the reason given in the plantar loading extraction rule: each region is a distinct anatomical quantity rather than a repeated measurement of one quantity.

The remaining studies that had supplied more than one dataset, Taspinar et al. [23] in the pain domain and Xu et al. [19] and Elsayed et al. [20] in the plantar pressure domain, are no longer represented more than once in any analysis, because each is withdrawn from, or not admitted to, the synthesis concerned on independent grounds reported in the Results. The violation itself is confirmed rather than explained away: it arose in the previous version, and it is now moot only because those studies no longer contribute. No study now contributes more than one estimate to any comparison. The single multiplicity that remains is the regional plantar loading profile, where it is anatomical rather than statistical, is displayed in full rather than reduced to a summary, and is not pooled.

#### 2.8.2 Sensitivity Analyses

Four sensitivity analyses were specified for this revision, and all four are reported in the Results whether or not they alter the interpretation. First, every synthesis was repeated with the two studies that did not allocate participants at random excluded, Taspinar et al. [23] and Khodaei et al. [24], so that the contribution of nonrandomized evidence to each domain can be read directly; where this exclusion empties a domain, that is stated rather than left to be inferred. Second, every paired contrast was recomputed with the assumed within‐participant correlation set to 0.3 and 0.7 as well as to 0.5 to display the full range of confidence‐interval widths the assumption can produce. Third, the pain estimate was recomputed on each of the other pain instruments reported by the contributing study, so that the dependence of the pain conclusion on the choice of instrument is quantified rather than assumed to be slight; because the pain domain now rests on a single instrument within a single study, this analysis is reported in the main text and not in a footnote. Fourth, the comparator‐stratum rule was relaxed and the estimate from stratum C was combined with the estimate from stratum A, to show what this review would have reported had that structural distinction not been drawn; the result is reported in full, including where it is narrower or more statistically significant than anything the review reports as its finding. In addition, final‐value and change‐from‐baseline analyses were compared for any contrast in which both were computable, and the comparison is reported wherever the two disagree.

### 2.9 Data Conversions

No included study reported a median or interquartile range for any outcome used in this review. Every outcome extracted was reported as a mean with a standard deviation, or as a mean with a standard error or 95% confidence interval from which the standard deviation was obtained directly. Therefore, no median‐to‐mean or interquartile‐range‐to‐standard‐deviation conversion was performed, and no sensitivity analysis comparing converted values with reported values was required.

### 2.10 Planned Subgroup and Meta‐Regression Analyses

Subgroup analyses by intervention type (custom vs. prefabricated) and by follow‐up duration, together with meta‐regression by intervention type and age group, were pre‐specified in the registered protocol. None of these analyses were performed. After the combinability conditions above are applied, a single study contributes to each outcome domain, which leaves nothing to compare between subgroups and is far below the minimum of approximately 10 studies per covariate recommended for meta‐regression [28]. The included populations also spanned children, adolescents, and adults, in some studies within a single sample, so participants could not be reliably assigned to age strata from the published reports. Intervention type, follow‐up duration, and age group were therefore considered narratively as potential sources of variation between studies. The narrative comparisons reported in the Discussion are exploratory, are not formal tests of effect modification, and should not be read as evidence that any of these factors does or does not modify the effect of orthoses.

## 3 Results

The systematic search yielded 290 articles. After removing 45 duplicates and screening 245 records, 59 articles underwent full‐text assessment. Eight studies met all inclusion criteria for final analysis, including a total of 382 unique participants with flexible flatfoot: Taspinar et al. [23] (*n* = 60), Yurt et al. [21] (*n* = 67), Elsayed et al. [20] (*n* = 40), Liebau et al. [18] (*n* = 52), Saeedi et al. [22] (*n* = 34), Yalfani et al. [17] (*n* = 30), Khodaei et al. [24] (*n* = 19), and Xu et al. [19] (*n* = 80). Two of these studies did not allocate participants at random: Taspinar et al. [23] reported that their three groups could not be randomized and assigned participants according to patient preference, and Khodaei et al. [24] used non‐random convenience sampling. Following independent re‐verification of every effect size, no outcome domain could be pooled. Each domain is now represented by a single study, and all pooled estimates, all heterogeneity statistics, and all summary diamonds reported in the previous version are withdrawn. Where a study contributed more than one eligible arm or more than one eligible measure within a domain, those data were either combined into one estimate or a single dataset was carried forward, so that no participant is now represented more than once within any analysis and the unit‐of‐analysis violation present in the previous version is removed. Publication bias could not be formally assessed using funnel plots or Egger's test due to insufficient studies (< 10) per outcome domain.

### 3.1 Pain Outcomes

No pooled pain estimate is reported. Of the four datasets that made up the previous pain pool, two were withdrawn on re‐verification, and the two that remain do not answer the same question: one compares an orthosis against a comparator providing no arch support, and the other compares an orthosis plus an adjunct exercise program against the same orthosis worn alone. They are therefore reported in separate strata, each containing a single study. With one estimate in each stratum, no pooled effect, no heterogeneity statistic, and no summary diamond can be computed, and no forest plot for pain is presented. Stratum A, orthosis versus a comparator providing no arch support, contains Yurt et al. [21] alone. On the 100 mm pain visual analog scale at 8 weeks, which the trial designated as its own primary outcome, the two active orthosis arms combined (mean 27.445, SD 17.431; *n* = 44) against the sham insole (mean 46.39, SD 20.18; *n* = 23) give *g* = −1.017 (SE 0.269; 95% CI −1.54 to −0.49). The two active arms were combined using the formula in Section 6.5.2.10 of the Cochrane Handbook [28], so that the shared sham arm is counted once and its 23 participants do not enter the analysis twice.

Stratum C, an orthosis combined with an adjunct exercise program versus the same orthosis worn alone, contains Elsayed et al. [20] alone. On the 0 to 10 numeric pain rating scale at 6 weeks, the combined arm scored 2.3 (SD 0.8) and the orthosis‐only arm 3.2 (SD 1.0), with 20 participants in each arm, giving *g* = −0.974 (SE 0.329; 95% CI −1.62 to −0.33). This estimate was relocated rather than withdrawn; it re‐verified exactly against the published report, and it is unchanged from the value given in the previous version. Because both arms wore the identical prefabricated insole, the contrast estimates the added effect of the short foot exercise program and not the effect of the orthosis. That is the reason it is held in its own stratum and is not combined with the stratum A estimate, and the reason it cannot be read as evidence about orthoses.

Taspinar et al. [23], which contributed two of the four datasets to the previous pain pool, is withdrawn on three independent grounds, the first of which is decisive on its own. The standard error of 0.215 published for a 20‐versus‐20 comparison is arithmetically impossible: the smallest standard error such a comparison can take, at a standardized mean difference of zero, is 0.3162, or 0.3099 after the Hedges correction [25]. The published value implies approximately 43 participants per arm rather than 20, and this conclusion rests on no assumption about which analysis the authors performed. Second, an exhaustive grid of 84 candidate computations (two instruments, two time points, all three pairwise contrasts, endpoint and change score at imputed correlations from 0.0 to 0.9, and change standardized by the baseline standard deviation) spans *g* from −2.379 to +2.379 and contains no value within 0.005 of either published figure, so neither reported effect size can be reproduced by any defensible route. Third, allocation was by participant self‐selection, the authors stating that they could not randomize their three groups, and baseline Foot Pain Index scores differed significantly across the arms (41.30, 30.35, and 23.25; *p* < 0.016). Endpoint and change‐score analyses of the same data consequently disagree in direction, giving +1.565 and +0.730 by endpoint against −1.246 and −0.385 by change score, so no analytic choice is neutral, and the trial carries a critical risk of bias on the ROBINS‐I confounding domain. Liebau et al. [18] contributed no dataset to the previous pool and contributes none now, for a simpler reason: the numeric rating scale it pre‐specified for pain is entirely unreported, so no pain estimate can be extracted from it at all.

One consequence of this stratification works against the review's own conclusion and should be stated plainly. Had the Elsayed et al. [20] estimate been admitted to stratum A alongside Yurt et al. [21], the two would have pooled to *g* = −1.000 (95% CI −1.41 to −0.59; I^2^ = 0%; *Z* = 4.80), an estimate both tighter and more strongly significant than anything reported in this review or in any previous version of it. It is nevertheless excluded from that pool, on a structural ground and not a statistical one: both Elsayed arms wore the orthosis, so that contrast cannot estimate the effect of the orthosis, however closely it happens to agree with Yurt et al. [21]. Numerical agreement between two contrasts does not make them measurements of the same thing. This is the only place in the manuscript where the forgone pooled figure is reported.

The stratum A estimate depends on which pain instrument is taken from Yurt et al. [21], and the size of that dependence is reported here rather than in a footnote. The pain visual analog scale, which the trial designated as its primary outcome, gives *g* = −1.017. The Foot Function Index total score, from the same trial, the same participants, and the same time point, gives *g* = −0.28, an effect that would be small and not distinguishable from no effect. The visual analog scale is used because the trial itself designated it as primary, which is a rule that can be specified in advance of seeing the data and applied without reference to the result it produces. It nonetheless remains the case that the entire pain conclusion of this review rests on one instrument choice within one trial, and it should be weighed accordingly.

### 3.2 Balance Outcomes

Balance and postural control are likewise reported from a single study, and no pooled estimate is presented. Yalfani et al. [17] measured the Biodex overall stability index at 12 weeks in 15 participants per arm. The orthosis group scored 0.93 (SD 0.60) and the control Group 1.46 (SD 0.60), giving *g* = −0.859 (SE 0.372; 95% CI −1.59 to −0.13). A lower index denotes better stability, so the estimate favors the orthosis, although its upper confidence limit (−0.13) lies close to no effect and the whole result rests on 30 participants in one trial. The static and dynamic plantar contact‐area data of Liebau et al. [18] were again not admitted to this domain, because contact area reflects loading distribution rather than postural stability.

Saeedi et al. [22] also measured a postural outcome, but it is reported narratively rather than combined with Yalfani et al. [17], because the two measurement conditions are opposite rather than merely different. Yalfani et al. [17] performed the 12‐week Biodex post‐test barefoot, with the orthosis removed, so that the estimate captures an adaptation effect persisting after the device is taken off. Saeedi et al. [22] measured center‐of‐pressure displacement in a single session with the device in situ, so that the estimate captures the immediate mechanical effect of wearing it. A summary combining an after‐removal adaptation effect with a while‐worn mechanical effect would correspond to no clinical question that could be asked of a patient, and the two were therefore not pooled. Narratively, Saeedi et al. [22] compared a University of California Biomechanics Laboratory (UCBL) orthosis and a modified UCBL foot orthosis (MUFO) with a shoe‐only condition in the same participants; the direction of the difference was not consistent across the gait phases reported, so the study is described rather than summarized as an effect. With one estimate in this domain, no heterogeneity statistic is reported and no forest plot for balance is presented.

### 3.3 Plantar Pressure Distribution

Four studies reported a plantar outcome, but no two of them contributed a comparable contrast and none could be pooled. Liebau et al. [18] reported static and dynamic plantar contact area and no pressure variable at all, so it contributes no loading estimate under the extraction rule stated in the Methods; its contact‐area values were also measured barefoot and are reported for a single placebo row that carries no separable comparator. Xu et al. [19] was not admitted to this domain, because its control arm wore a prefabricated foot orthosis: that contrast sets one orthosis against another and answers a different question, and the cell that would have contributed was also imbalanced at baseline. Elsayed et al. [20] was not admitted, because both of its arms wore the identical prefabricated insole, so its between‐group difference estimates the added effect of short foot exercise and no effect of the device on loading. Only Khodaei et al. [24] compared loading with an orthosis against loading without one, and this domain therefore rests on that single study (*k* = 1). No pooled estimate, no heterogeneity statistic, and no summary diamond are reported for plantar loading. Khodaei et al. [24] measured 19 participants in every condition, so the comparison is within‐subject; the two orthosis conditions were combined and a correlation of 0.5 between paired conditions was assumed. The studies contributing an estimate enrolled adolescents and adults; the only trial of children contributed no estimate, so the evidence could not be stratified by age. Eight‐foot regions were reported separately, and they are given separately here (Table 1, Figure 2).

**TABLE 1:** Region‐specific standardized mean differences for plantar loading with an orthosis in Khodaei et al. [24] (*n* = 19; within‐subject, each participant measured in every condition; the two orthosis conditions combined; correlation between paired conditions assumed to be 0.5).

Plantar region	Hedges' g (95% CI)
Second metatarsal	−1.928 (−2.68 to −1.18)
Third‐to‐fifth metatarsals	−1.319 (−1.92 to −0.72)
Heel	−1.089 (−1.64 to −0.54)
Lateral midfoot	−0.471 (−0.93 to −0.01)
Hallux	−0.413 (−0.86 to +0.04)
First metatarsal	−0.178 (−0.61 to +0.26)
Lateral toe	+0.599 (+0.13 to +1.07)
Medial midfoot	+1.756 (+1.05 to +2.46)

*Note:* Negative values denote reduced loading under the orthosis, positive values increased loading. Regions are ordered from the largest reduction to the largest increase.

**FIGURE 2:**
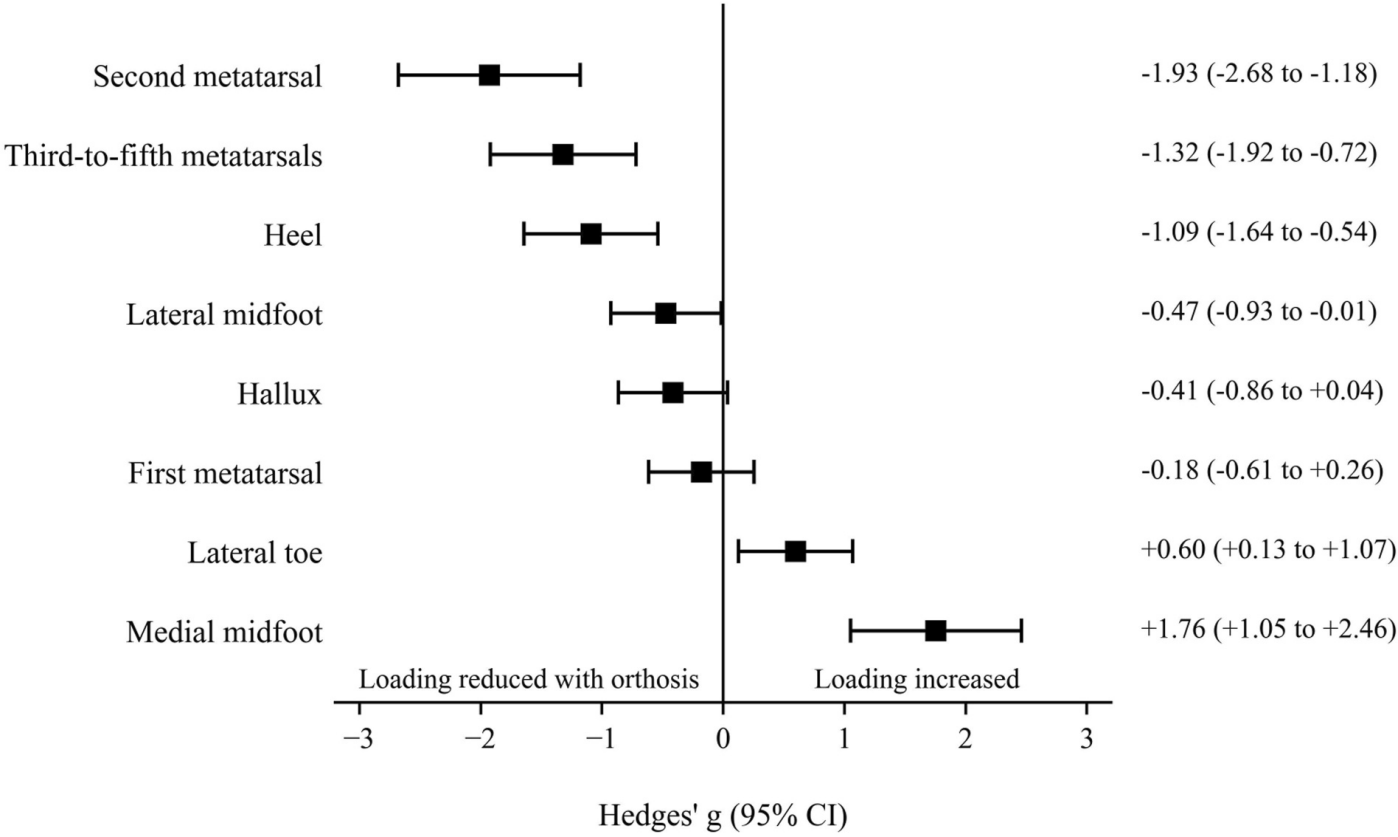
Region‐specific standardized mean differences for plantar loading under an orthosis in Khodaei et al. [24], by foot region.

The eight regional estimates do not share a direction, and that is the finding. Loading fell most at the second metatarsal (*g* = −1.928), at the third‐to‐fifth metatarsal (*g* = −1.319), and at the heel (*g* = −1.089), with confidence intervals excluding the null in all three. Loading rose at the medial midfoot (*g* = +1.756) and at the lateral toe (*g* = +0.599), again with confidence intervals excluding the null. Of the remaining three regions, the reduction at the lateral midfoot (*g* = −0.471, 95% CI −0.93 to −0.01) reached the null boundary and should be read as borderline, while the reductions at the hallux (*g* = −0.413) and the first metatarsal (*g* = −0.178) had confidence intervals crossing the null. The load therefore shifted away from the heel and the lesser metatarsals and toward the medial midfoot, which is the mechanical effect that a medial arch support is built to produce. A single pooled figure is not reported for this domain because averaging these regions would set reductions against increases of comparable magnitude and return a value near zero, concealing the redistribution rather than describing it. Because the design is within‐subject, the estimates are paired and rest on an assumed correlation of 0.5 between conditions; a different assumed correlation would widen or narrow all eight intervals together and shift the point estimates slightly, because the two orthosis conditions are combined before the contrast is taken, without altering their relative ordering or the direction of the redistribution (Figure 2).

### 3.4 Co‐Interventions Across Included Trials

Co‐interventions differed across the included studies, and the distinction between balanced and unbalanced co‐interventions is relevant to the interpretation of every estimate reported above. Yurt et al. [21] and Liebau et al. [18] delivered the same exercise program to every arm, so the exercise component was balanced across groups and does not confound the orthosis contrast in those trials. In Taspinar et al. [23], the exercise program was not a co‐intervention but the comparator arm itself, withheld from both device arms; only disease education and monthly outpatient review were common to all three arms. Elsayed et al. [20] was the only trial with an unbalanced co‐intervention: an orthosis was worn in both arms, and the experimental arm additionally performed short foot exercises, so its between‐group estimate reflects the incremental effect of the exercise program added to an orthosis rather than the effect of the orthosis alone. Yalfani et al. [17], Saeedi et al. [22], Khodaei et al. [24], and Xu et al. [19] delivered no co‐intervention, so their contrasts are uncontaminated by concurrent treatment, although in Xu et al. [19] the control arm itself wore a prefabricated orthosis, which makes that trial a comparison of one device with another rather than of a device with none. Study‐level details on populations, orthosis type, co‐interventions, and follow‐up duration are provided in Supporting Information S2.

### 3.5 Clinical Significance of Effect Sizes

Statistical significance alone does not establish clinical relevance, and the four results reported above differ in how they should be read against a clinical threshold. For pain, the registered protocol defined the minimal clinically important difference as at least 2 points on a 10‐point pain scale, equivalent to 20 mm on a 100 mm visual analog scale. In Yurt et al. [21], the between‐group difference on the 100 mm pain visual analog scale was 18.95 mm, which approaches but does not reach that threshold, and the corresponding standardized effect of *g* = −1.017 is large. That reading depends on which of the trial's own pain instruments is used: on its Foot Function Index total the same trial gives *g* = −0.28, a small effect. The magnitude of the pain finding in this review is therefore not a property of the evidence base but of a single instrument choice within a single trial, and it should be read with that in view. In Elsayed et al. [20], the difference of 0.9 points on a 0‐10 scale (2.3 vs. 3.2) is well below the 2‐point threshold, and it is attributable to the added short foot exercise rather than to the device, since both arms wore the same insole. For balance, no consensus minimal clinically important difference exists for the Biodex overall stability index; the estimate of *g* = −0.859 is large by conventional benchmarks, but its confidence interval reaches −0.13, and whether the effect translates into patient‐relevant outcomes cannot be judged, because no included study recorded falls, injuries, or activity limitation. For plantar loading, the regional estimates are large, but they describe where load is carried rather than how much a patient hurts; no included study related the magnitude or the direction of pressure change to change in symptoms, so the observed redistribution should not be assumed to deliver a symptomatic benefit.

### 3.6 Sensitivity Analyses

The four sensitivity analyses specified in the Methods are reported here in full. Excluding the two studies that did not allocate participants at random changes nothing in the pain domain, where Taspinar et al. [23] is already withdrawn on the arithmetic ground given above, and nothing in the balance domain, which rests on a randomized trial. It empties the plantar loading domain completely, because Khodaei et al. [24] is the only study admitted to it. The regional redistribution described above is therefore supported by nonrandomized evidence alone, and a reader who declines to accept nonrandomized evidence in this review is left with no plantar loading finding at all. This is stated rather than left to be inferred.

Varying the assumed within‐participant correlation across 0.3, 0.5 and 0.7 slightly moves every regional estimate and not only its confidence interval. The correlation enters the standard deviation obtained when the two orthosis conditions are combined, and that standard deviation is part of the standardizer, so the point estimate moves with it. The movement is small: no region shifts by more than 0.13 on the standardized scale across that range, and the direction and the rank order of the eight regions are the same at every value. The second metatarsal runs from −1.959 (95% CI −2.85 to −1.06) at 0.3 to −1.898 (−2.47 to −1.32) at 0.7, and the medial midfoot runs from +1.820 (+0.97 to +2.67) to +1.699 (+1.16 to +2.23). Those two regions, together with the third‐to‐fifth metatarsals, the heel and the lateral toe, exclude the null at every assumed correlation. None of the three small regional effects does, and the point at which each turns is close enough to the assumed value to be stated exactly. The lateral midfoot excludes the null only where the assumed correlation exceeds 0.465, so its significance at 0.5 is produced by the assumption rather than by the data; at 0.5 its upper limit is −0.01. The hallux excludes the null only above 0.586, so it does not at 0.5 but does at 0.7. Neither is offered as a finding for that reason. The first metatarsal would require an assumed correlation above 0.925 and includes the null throughout.

The third and fourth sensitivity analyses are reported at the point where each bears on the result: the dependence of the pain estimate on the choice of instrument within Yurt et al. [21], and the pooled figure that relaxing the comparator‐stratum rule would have produced, are both given in the pain section above. Final‐value and change‐from‐baseline analyses were compared wherever both were computable. They agree in every contrast reported in this review and disagree only in Taspinar et al. [23], where the two approaches return effects of opposite sign, which is one of the grounds for that study's withdrawal.

### 3.7 Risk of Bias Assessment

Risk of bias was assessed with RoB 2 for the six studies that randomly allocated participants. One was rated at low risk of bias (Yalfani et al. [17]) and five at some concerns (Yurt et al. [21], Xu et al. [19], Elsayed et al. [20], Liebau et al. [18], Saeedi et al. [22]); none was rated at high risk (Figure 3). The rating for Yurt et al. [21] is given in full here, because that trial carries the pain estimate this review reports. Its report states that the randomization sequence was regenerated part‐way through the trial, after each group had reached at least 10 participants, because baseline pain in one arm was running high; the balance of the final groups is therefore a product of that intervention rather than of an untouched random sequence, and the first domain is rated at some concerns on that ground. Its report also states that most participants were aware that the sham insole was not a supportive one, so blinding of participants was not maintained, and the second domain is rated at some concerns on that ground. Because RoB 2 presupposes randomized allocation and does not assess confounding factors, the two studies that did not randomize were appraised with ROBINS‐I instead, as the registered protocol specified for nonrandomized studies (Figure 4). Taspinar et al. [23] was rated critical risk on the confounding domain: participants selected their own group, and baseline Foot Pain Index scores differed significantly across arms (41.30, 30.35, and 23.25; *p* < 0.016). Khodaei et al. [24] were rated at serious risk of bias overall. Its within‐participant design removes confounding by participant characteristics, since every participant was measured under every condition, so the confounding domain is not the difficulty there that it is in Taspinar et al. [23]. The report does not state that the order of the three conditions was randomized or counterbalanced, participants were a convenience sample, and twenty‐four region‐by‐parameter cells are reported with no registered protocol against which the selection of the reported result could be checked.

**FIGURE 3:**
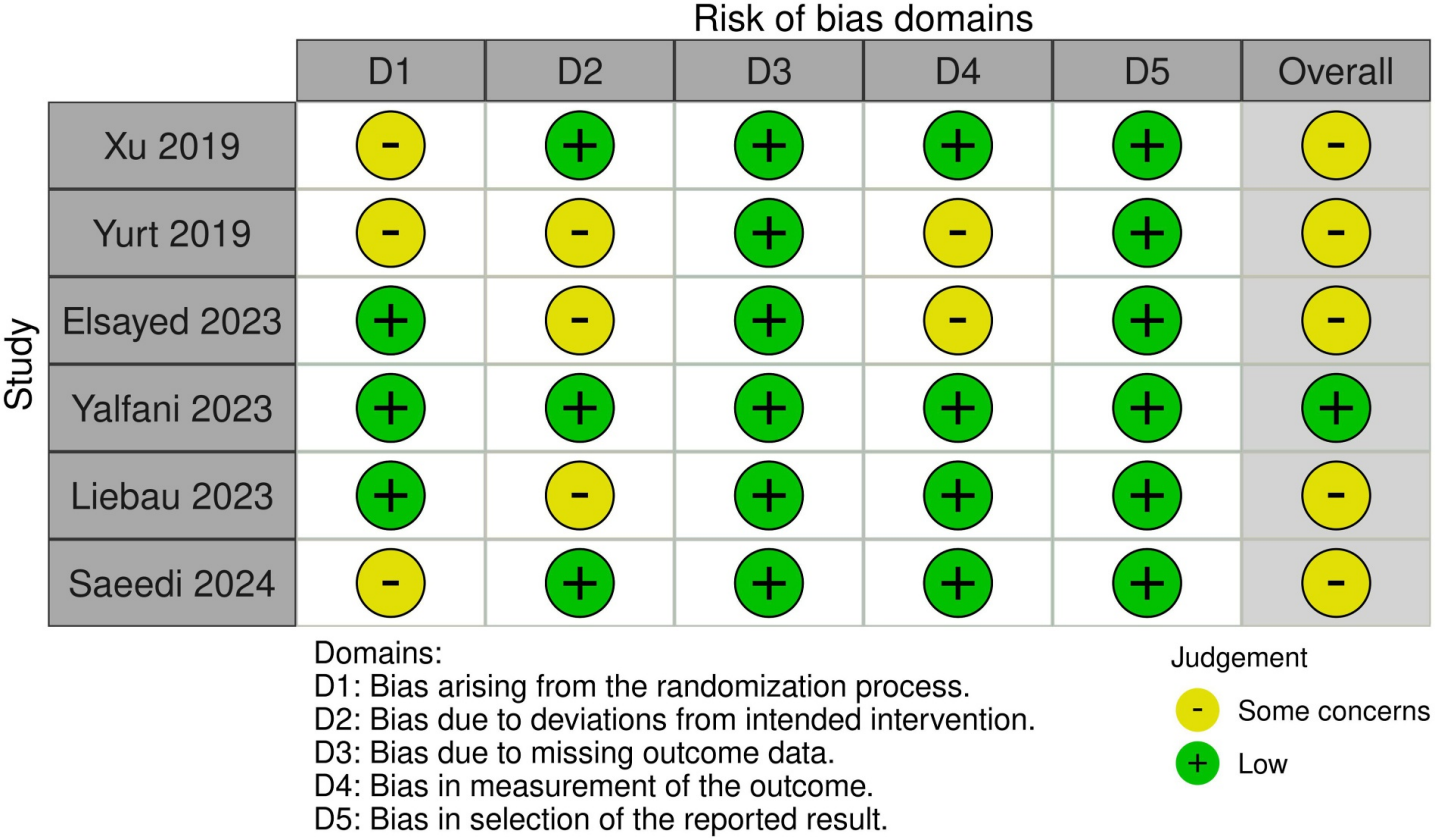
Summary of RoB 2 assessment for orthotic intervention studies in flexible flatfoot.

**FIGURE 4:**
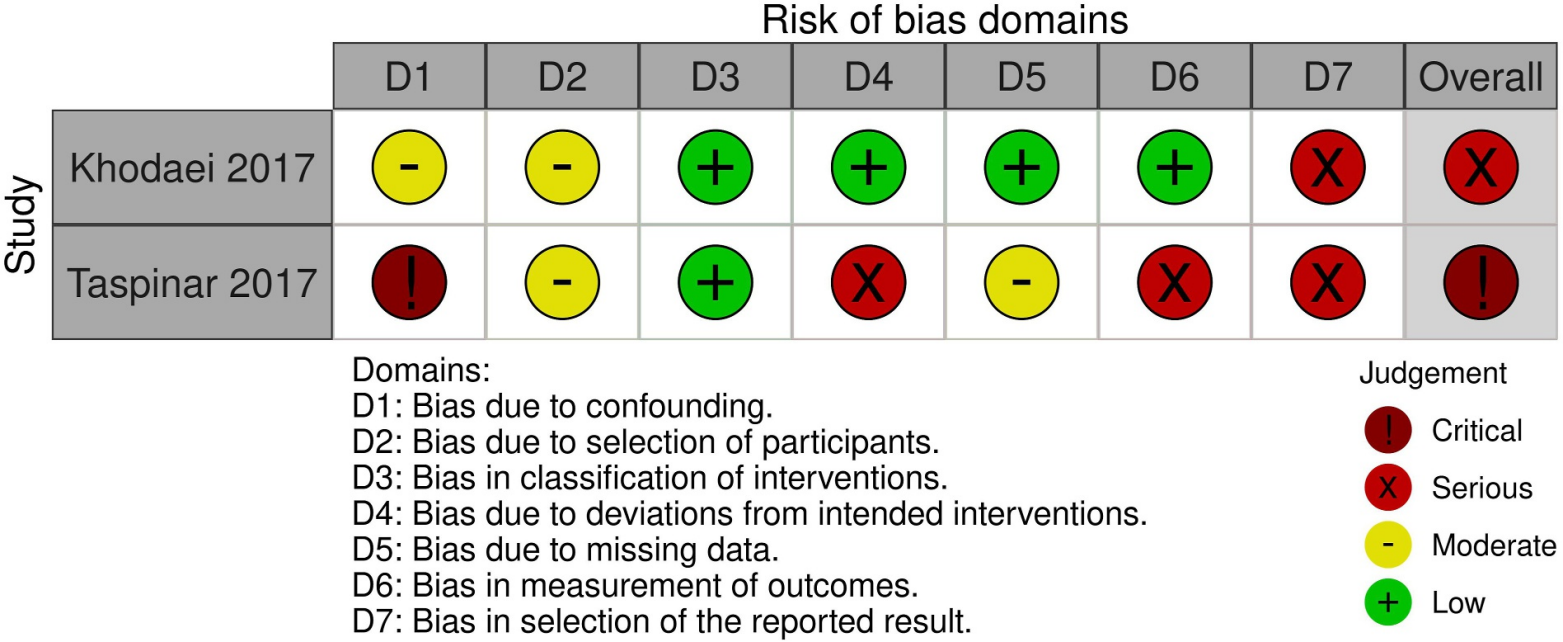
Summary of ROBINS‐I assessment for the two studies that did not allocate participants at random.

Across the six studies assessed with RoB 2, the domains covering missing outcome data and selection of the reported result were rated as low risk throughout. Measurement of the outcome was rated at low risk in four, each of which measured its outcome with an instrument; it was rated at some concerns in Yurt et al. [21] and Elsayed et al. [20], whose outcomes were participant‐reported pain scales and whose participants were not blinded, so that the person reporting the outcome knew which intervention had been received. Three studies were rated at some concerns on the randomization process, Yurt et al. [21], Xu et al. [19] and Saeedi et al. [22], and three on deviations from intended interventions, Yurt et al. [21], Elsayed et al. [20] and Liebau et al. [18] (Figure 3).

The certainty of evidence was rated using the GRADE approach [32] (Table 2). Because no outcome domain could be pooled, certainty was rated separately for each of the four rows of that table rather than for a body of pooled evidence, and inconsistency was not rated in any domain, since consistency cannot be assessed where only one study contributes. Pain against a comparator providing no arch support was rated very low certainty, downgraded for imprecision (one trial of 67 participants, with the optimal information size not met), for risk of bias arising from selection of the reported result (the trial reported more than one pain instrument, and the estimate moves from *g* = −1.017 on the visual analog scale it designated as its primary outcome to *g* = −0.28 on its Foot Function Index total), and for publication bias, which cannot be excluded where a domain rests on one small trial. Pain for short foot exercise added to an orthosis was also rated very low certainty, downgraded for risk of bias (Elsayed et al. [20] was rated “some concerns” on RoB 2), for imprecision (one trial of 40 participants), and for publication bias. Balance was rated very low certainty, downgraded for imprecision (one trial of 30 participants, with a confidence interval reaching −0.13), for indirectness (the 12‐week post‐test was performed barefoot with the orthosis removed, so it measures an adaptation effect rather than the effect of wearing the device, and the participants were competitive taekwondo athletes), and for publication bias. Plantar loading began at low certainty because the contributing study did not randomize allocation, and was further downgraded for risk of bias, for imprecision (19 participants), and for indirectness (a single‐session surrogate measure, analyzed under an assumed correlation between paired conditions, that no included study linked to symptoms). Therefore, certainty is very low for every outcome in this review, and no estimate should be read as a stable magnitude. Follow‐up was predominantly short term, and publication bias could not be formally assessed in any domain, because each domain rests on a single study.

**TABLE 2:** Summary of findings: orthotic interventions for flexible flatfoot, by outcome and comparator.

Orthotic interventions for flexible flatfoot: Single‐study estimates, by outcome and comparator						
Patient or population: People with flexible flatfoot Setting: Clinical research centers Intervention: Foot orthosis, insole, or arch support Comparison: Varies by outcome and is stated in each row: a sham insole providing no arch support, no orthosis, the same participants measured without a device, or the same orthosis worn in both arms						

**Outcome № of participants (studies)**	**Relative effect (95% CI)**	**Anticipated absolute effects (95% CI)**			**Certainty**	**What happens**
		**Comparator**	**Orthosis**	**Difference**		
Balance and postural control (postural stability) Assessed with: Biodex overall stability index, endpoint at 12 weeks, measured barefoot with the orthosis removed Comparator: No orthosis Follow‐up: 12 weeks № of participants: 30 1 trial (Yalfani et al. [17])	—	—	—	SMD 0.86 SD lower (1.59 lower to 0.13 lower)	⨁◯◯◯ Very low a, b, c	The evidence for balance is very uncertain. One trial contributes. In taekwondo athletes with flexible flatfoot, the Biodex overall stability index at 12 weeks was 0.93 with an orthosis and 1.46 without one (*g* = −0.859, *P* = 0.02). The post‐test was performed barefoot with the orthosis removed, so this is an adaptation effect after 12 weeks of wear rather than the effect of the device while it is worn. No consensus minimal clinically important difference exists for this index, and none of the included studies measured falls, injuries, or activity limitation directly.
Plantar loading, by foot region (pressure redistribution) Assessed with: regional plantar pressure, eight foot regions, single session Comparator: The same participants measured without an orthosis Follow‐up: single session № of participants: 19 1 study (Khodaei et al. [24]), within‐subject	—	—	—	No single estimate Region‐specific SMDs range from 1.93 SD lower at the second metatarsal (2.68 lower to 1.18 lower) to 1.76 SD higher at the medial midfoot (1.05 higher to 2.46 higher); all eight regions are given in Table 1	⨁◯◯◯ Very low a, d, e	Orthoses redistribute plantar load rather than lowering it uniformly. Loading fell at the second metatarsal, at the third‐to‐fifth metatarsals, and at the heel, and rose at the medial midfoot and the lateral toe, which is the mechanical effect a medial arch support is designed to produce. A single averaged figure would set these reductions against the increases and approach zero, concealing the redistribution, so none is reported. The evidence is very uncertain: One small study that did not randomize allocation contributes, and no study in this review related the size or the direction of pressure change to change in symptoms.
Pain (pain reduction), against a comparator providing no arch support Assessed with: 100 mm pain visual analog scale at 8 weeks, the trial's designated primary outcome Comparator: Sham insole Follow‐up: 8 weeks № of participants: 67 1 trial (Yurt et al. [21]), the two active orthosis arms combined	—	—	—	SMD 1.02 SD lower (1.54 lower to 0.49 lower)	⨁◯◯◯ Very low a, c, f	The evidence for pain reduction is very uncertain. One trial contributes. On its designated primary outcome, a 100 mm pain visual analog scale at 8 weeks, orthoses were 1.02 standard deviations better than a sham insole (*P* < 0.001), a between‐group difference of 18.95 mm that approaches but does not reach the predefined minimal clinically important difference of 20 mm. The estimate is not robust to the choice of instrument within that same trial: Its foot function index total gives *g* = −0.28, a small effect. The pain conclusion of this review therefore rests on a single instrument choice within a single trial, and it should not be read as a stable estimate of how much orthoses reduce pain.
Pain (pain reduction), for short foot exercise added to an orthosis Assessed with: numeric pain rating scale, 0–10, at 6 weeks Comparator: The same prefabricated insole worn without the exercise program Follow‐up: 6 weeks № of participants: 40 1 trial (Elsayed et al. [20])	—	—	—	SMD 0.97 SD lower (1.62 lower to 0.33 lower)	⨁◯◯◯ Very low a, c, g	This row does not estimate the effect of an orthosis. Both arms wore the identical prefabricated insole and only one performed short foot exercises, so the contrast is the added effect of the exercise program: 2.3 versus 3.2 points on a 0‐10 scale at 6 weeks (*P* = 0.003), a difference of 0.9 points, below the predefined 2‐point threshold. It is reported in a separate row rather than combined with the row above. That separation is not costless, and the pooled figure it forgoes is reported in full in the results; it is kept separate anyway, because a contrast in which both arms wore the orthosis cannot contribute to an estimate of what the orthosis does.

*Note:* All outcomes in this table are continuous, so no relative effect and no anticipated absolute risk can be calculated, and those columns are marked with a dash. Every row rests on a single study, so no pooled estimate, no heterogeneity statistic, and no summary diamond is reported. Four eligible studies contribute no row: Taspinar et al. [23] and Liebau et al. [18] yielded no usable pain estimate, Saeedi et al. [22] measured center‐of‐pressure with the device in situ in a single session and is reported narratively, and Xu et al. [19] compared one orthosis with another. The grounds are given in the Results.

Abbreviations: CI, confidence interval; SMD, standardized mean difference.

GRADE Working Group grades of evidence. Inconsistency was not rated in any row, because a single study contributes to each outcome and consistency cannot be assessed. Explanations: (a) Downgraded one level for imprecision: the outcome rests on a single study and the optimal information size is not met. (b) Downgraded one level for indirectness: the 12‐week post‐test was performed barefoot with the orthosis removed, so it measures an adaptation effect rather than the effect of wearing the device, and the participants were competitive taekwondo athletes. (c) Downgraded one level for publication bias: single small study contributes, replication is absent, and small‐study effects cannot be excluded. (d) Certainty began at low rather than high because the contributing study did not randomize allocation, using non‐random convenience sampling, and it was rated at serious risk of bias on ROBINS‐I [27]. (e) Downgraded one level for indirectness: loading was measured in a single session as a within‐subject comparison analyzed under an assumed correlation of 0.5 between paired conditions, and no included study related the magnitude or the direction of pressure change to change in symptoms. (f) Downgraded one level for risk of bias. The contributing trial is rated at some concerns on the RoB 2 domains covering the randomization process and deviations from intended interventions, for the reasons given in the Results, and there is a further concern in the selection of the reported result: the trial reported more than one pain instrument, and the estimate moves from *g* = −1.017 on the visual analog scale the trial designated as its primary outcome to *g* = −0.28 on its Foot Function Index total. The visual analog scale was selected because the trial designated it, which is a pre‐specifiable rule, but the conclusion is not robust to that choice. (g) Downgraded one level for risk of bias: the contributing trial was rated “some concerns” on the RoB 2 assessment.

## 4 Discussion

This systematic review evaluated orthotic interventions for flexible flatfoot across three outcome domains. The independent re‐verification undertaken for this revision changed what the review is able to report. No outcome domain retains two or more compatible datasets, so every pooled estimate and every heterogeneity statistic has been withdrawn. What remains is a small set of single‐study estimates, each answering a narrower question than a pooled effect would. Pain is represented by one orthosis‐versus‐sham comparison and, separately, by one orthosis‐plus‐exercise comparison. Balance rests on one trial, and plantar loading on one within‐subject study reported region by region. The evidence base is therefore thinner than the previous version implied, and it is discussed here study by study.

Pain is now reported in two strata, because the two available contrasts are not commensurable. In the first stratum, an orthosis is compared with a comparator that provides no arch support. Only Yurt et al. [21] supply such a contrast, and its estimate is large (*g* = −1.017, 95% CI −1.54 to −0.49). The outcome is the 100 mm pain visual analog scale at 8 weeks, which that trial designated as its primary outcome. The two active orthosis arms were combined into one group using the Cochrane Handbook formula for independent parallel arms [28]. The combined group scored 27.445 (SD 17.431) in 44 participants, against 46.39 (SD 20.18) in 23 recipients of a sham insole. All 67 randomized participants were counted exactly once. On the original scale, the difference was 18.95 mm, short of the 20 mm minimal clinically important difference set in the protocol.

This single estimate is sensitive to one instrument choice, and that sensitivity belongs to the main text. Yurt et al. [21] also reported the Foot Function Index. On the pain visual analog scale, the effect is *g* = −1.02; on the foot function index total, it is *g* = −0.28. The visual analog scale is used here because the trial designated it as primary, which is a rule that can be specified in advance. It was not chosen because it yields a larger number. Even so, the pain conclusion for this stratum turns on that one decision, inside one trial of 67 participants. The direction of effect is better supported than its magnitude, and the magnitude should be treated as unstable.

The second stratum asks a different question. Elsayed et al. [20] gave both arms an identical prefabricated insole and added short foot exercise to one arm only. That contrast estimates the added value of the exercise, not the effect of the orthosis. The estimate is large (*g* = −0.974, 95% CI −1.62 to −0.33), measured on a 0‐10 pain rating scale after 6 weeks. Scores were 2.3 (SD 0.8) against 3.2 (SD 1.0), with 20 participants per arm. This value was re‐verified exactly on independent recalculation. It has been relocated to its own stratum rather than withdrawn, because the estimate is sound and only its pooling was wrong. Combining it with the stratum A estimate would produce a tighter and more significant pooled figure, which is reported in full in the Results, but a contrast in which both arms wore the orthosis cannot contribute to an estimate of what the orthosis does.

Taspinar et al. [23] has been withdrawn from the pain synthesis on three independent grounds, as set out in full in the Results. Its published standard error of 0.215 is arithmetically impossible for a comparison of 20 against 20, where the smallest attainable value is 0.3162, or 0.3099 after the Hedges correction. No computation among the 84 candidates enumerated reproduces any published figure. Allocation was by participant preference rather than at random, with baseline Foot Pain Index scores differing across the arms, so that endpoint and change‐score analyses of the same data reverse the direction of effect. The study is rated at a critical risk of bias on the ROBINS‐I confounding domain and contributes no numeric estimate. Liebau et al. [18] contribute none either because its pre‐specified numeric rating scale is entirely unreported.

Balance now rests on a single study. Yalfani et al. [17] reported the Biodex overall stability index at 12 weeks, with 15 participants per arm. Scores were 0.93 (SD 0.60) with the orthosis against 1.46 (SD 0.60) in the control arm, giving *g* = −0.859 (95% CI −1.59 to −0.13). The interval is wide and its upper limit lies close to the null, so the size of any benefit is poorly determined. A lower stability index indicates a steadier posture, so the direction favors the orthosis. Earlier, syntheses of orthoses and insoles on postural balance reported effects in the same direction, although in different populations and with different devices [33, 34, 35].

Saeedi et al. [22] was previously pooled with this trial, and that pooling was an error of kind rather than of arithmetic. The two studies measured under opposite conditions. Yalfani et al. [17] tested barefoot at 12 weeks with the orthosis removed, so the measurement captures adaptation that persists without the device. Saeedi et al. [22] measured center of pressure in a single session with the device in situ, capturing an immediate mechanical effect. One asks whether the foot has changed and the other asks what the device does while it is worn. Averaging the two answers neither question, so Saeedi et al. [22] is now reported narratively and contributes no pooled weight.

The plantar pressure pool has also been withdrawn, and this withdrawal produced the most informative result in the review. The previously reported pooled value averaged several foot regions and three studies into one number. That number described a general lowering of pressure. It could not describe where the load went, which is the only question a medial arch support actually poses. Xu et al. [19] are no longer admitted because their control arm also wore a prefabricated orthosis. That contrast compares two devices rather than one against none, and its selected cell was imbalanced at baseline. Elsayed et al. [20] raised the same structural problem in this domain, since both of its arms wore the identical insole.

Khodaei et al. [24] is therefore reported alone, and as eight region‐specific estimates rather than one. The design is within‐subject, with 19 participants and the orthosis conditions combined, assuming a within‐person correlation of 0.5. Peak pressure fell markedly under the second metatarsal (*g* = −1.928, 95% CI −2.68 to −1.18). It fell under the third‐to‐fifth metatarsals (*g* = −1.319, −1.92 to −0.72) and under the heel (*g* = −1.089, −1.64 to −0.54). A smaller reduction at the lateral midfoot only just excluded the null (*g* = −0.471, −0.93 to −0.01), and a comparable reduction at the hallux did not (*g* = −0.413, −0.86 to +0.04). The first metatarsal barely changed (*g* = −0.178, −0.61 to +0.26). Two regions took on more load: the lateral toe (*g* = +0.599, +0.13 to +1.07) and, most of all, the medial midfoot (*g* = +1.756, +1.05 to +2.46). Varying the assumed correlation between 0.3 and 0.7 moves each estimate by less than 0.13 on the standardized scale and changes neither the direction nor the ordering of the eight regions.

Taken together, these eight estimates describe a redistribution of load rather than a reduction of it. Load moves away from the heel and the lesser metatarsals, and toward the medial midfoot. That is precisely the mechanical action a medial arch support is built to perform. The support occupies the space under the medial longitudinal arch, accepts load there, and unloads the structures at either end of the foot. The two largest effects anywhere in this review are the medial midfoot increase and the second metatarsal decrease, and they point in opposite directions. A single pooled figure averages them toward zero and conceals the mechanism entirely. Its magnitude would also depend on how many regions happened to be extracted, which is not a property of the intervention. This mechanical account is consistent with earlier work on how orthoses act on foot loading and on gait [36, 37].

Two cautions attach to this finding. It comes from one small nonrandomized study measured in a single laboratory session, so it describes a mechanism rather than establishing a benefit. No included study related the size of a regional pressure change to a change in symptoms. A redistribution of this kind is therefore consistent with the design intent of the device, without providing evidence that patients feel better for it. Future plantar pressure trials should report region by region for this reason.

### 4.1 Clinical Implications

These findings support a narrower claim than the previous version made. Each domain now rests on one study, so orthoses cannot be recommended here on the strength of pooled effects. What the corrected evidence supports best is a mechanical account of how the device acts. Orthoses change where the foot is loaded, and one study documents that change in regional detail. Effects on pain and on balance are each supported by a single trial with a wide confidence interval. Individualized assessment therefore matters more rather than less, because no average effect is available to guide prescription [38].

Clinicians should also note that no domain has independent replication. A single study cannot show whether a result generalizes beyond its own sample, protocol, and device. Age was considered only narratively, so age‐specific recommendations cannot be drawn from this evidence. Pain response may still depend on activity level, body mass index, and pain characteristics [39]. That expectation is now a hypothesis for future trials rather than an inference from these data. Assessment should still combine structural measurement with functional evaluation of balance and plantar loading.

No comparison of custom devices against prefabricated devices can be supported by this review. Pre‐specified subgroup analysis was not performed, and each domain now contains one study. Khodaei et al. [24] did compare a computer‐aided device with a prefabricated one, but its two conditions were combined for the analysis reported here. Xu et al. [19] made a similar device‐against‐device comparison and it is not admitted to the plantar stratum. Work outside this review suggests that orthosis design parameters have measurable dose‐dependent effects on loading and lower‐limb kinematics [40, 41], but the present studies cannot quantify them. Cost, availability, and patient preference should therefore weigh at least as heavily as the manufacturing route. Clear wearing protocols and an adaptation period remain sensible practice, not findings of this review.

From a rehabilitation perspective, the best documented action of an orthosis is on the distribution of foot loading. Yurt et al. [21] and Yalfani et al. [17] each add one estimate, for pain and for balance, and each stands alone. Orthoses should therefore be offered as one component of care rather than as a stand‐alone treatment. Combination with exercise has the most direct support available here. Elsayed et al. [20] added short foot exercise to an orthosis worn by both arms, and pain fell further (*g* = −0.974). That trial isolates the exercise, which is exactly why it cannot also serve as evidence for the orthosis. Prefabricated devices remain a reasonable first option where cost or access is a barrier, on practical grounds rather than on comparative evidence.

### 4.2 Limitations of the Evidence Base

Several limitations of the evidence base are now more serious than the previous version stated. First, no outcome domain retains two or more compatible datasets, so nothing in this review can be pooled. Every estimate reported here comes from a single study, and none has independent replication. Second, the contributing studies are small. The largest contributing sample is 67 participants, and the plantar loading analysis rests on 19. Third, two of the eight studies did not allocate participants at random. Taspinar et al. [23] assigned participants by preference and Khodaei et al. [24] used convenience sampling, so confounding cannot be excluded in either. The plantar loading result depends on one of these two studies.

Fourth, one trials published effect sizes could not be reconstructed at all. The entries reported by Taspinar et al. [23] cannot be reproduced from its own source tables by any of 84 candidate computations. Its published standard error is also arithmetically unattainable at its stated sample size. Effect sizes that cannot be recovered from the source cannot be verified by any reader, and cannot enter a synthesis. Fifth, the two balance studies were measured under fundamentally different conditions, one barefoot after 12 weeks of wear and one with the device in place in a single session. These are not two measurements of a single construct, and no analytic remedy makes them one.

Sixth, one trial's pain data are simply missing from the literature. Liebau et al. [18] pre‐specified and measured pain on a numeric rating scale in 52 children, in a randomized double‐blind sham‐controlled trial, and published no value for it. Selective non‐reporting of this kind cannot be detected by funnel plots and is not corrected using any statistical method. Seventh, the comparators differ fundamentally across studies, from a sham insole, to an alternative device, to the same device worn in both arms. Each contrast answers a different clinical question, which is why the strata are kept apart. Eighth, follow‐up of the contributing estimates ran from a single session to 12 weeks, so durability beyond 3 months is untested; the only longer follow‐up in the review, at 6 and 12 months in Liebau et al. [18], contributes no estimate. Finally, participants cannot be blinded to a device that is worn and felt [41], which leaves self‐reported pain open to expectation effects.

### 4.3 Limitations of the Review Process

Limitations of the review process itself should be considered separately, and the most important of them is ours. The pooled estimates in the previous version rested on unit‐of‐analysis violations. Four studies each contributed two or more datasets to a single pool, so the same participants were counted more than once in one analysis. That practice overstates precision and misstates the weight carried by individual studies. It also concealed the fact that datasets within a pool were sometimes answering different questions. The violation has been corrected by dissolving the pools rather than by adjusting them because after correction, no domain retains enough compatible datasets to pool. Two extraction errors identified in earlier rounds prompted this re‐verification, and the re‐verification changed the conclusions of the review.

A second limitation of the process concerns the rules used to replace those pools. The extraction and stratification rules applied here were written during revision, after the candidate data cells had been inspected. They were not part of the registered protocol and cannot be described as pre‐specified. They are presented instead as rules that could have been specified in advance from design and construction premises alone. The same team that identified the defects also wrote the replacement rules, without independent statistical review. The mitigation adopted is full disclosure: every rejected alternative is reported with its value, so that a reader who disagrees can compute a different answer.

The limitations of the search stand as previously described. Three databases were searched, and eligibility was restricted to full‐text reports in English, so language bias cannot be excluded. Reference lists were not screened and no citation tracking was performed although reference‐list screening had been specified in the registered protocol. Neither clinical‐trial registries nor grey‐literature sources were searched, and eligible studies may therefore have been missed. Publication bias could not be assessed, because no domain now contains more than one study. Funnel plots and Egger's test are not interpretable at that number, and small‐study effects cannot be excluded. Pre‐specified subgroup and meta‐regression analyses could not be performed and were considered only narratively. Screening was independent and in duplicate, but reviewers were not blinded to authorship, institution, or journal.

### 4.4 Future Directions

Future research should make trials comparable enough to be combined. Reports should give between‐group means, standard deviations, and exact group sizes at every time point, so that effect sizes can be reconstructed independently. Designating and reporting one primary pain instrument would remove the instrument sensitivity seen here. Comparators should be defined explicitly, ideally including a true no‐orthosis or sham arm, so that the device can be separated from co‐interventions. Plantar pressure should be reported region by region, because regional redistribution is the mechanism of interest and a single pooled figure hides it. Balance protocols should state whether measurement occurs with the device in place or after its removal, since the two answers different questions. Longer follow‐up is needed to test durability beyond 12 weeks. Randomization and its reporting should improve, given that two included studies did not randomize allocation [42]. A sufficient body of comparable trials would then permit the subgroup and meta‐regression analyses that could not be attempted here. An individual participant data meta‐analysis would be the most efficient route, and would allow correlated datasets within a trial to be handled properly.

## 5 Conclusion

This systematic review found that the available studies cannot be pooled. Independent re‐verification withdrew every pooled estimate reported in the previous version, and each outcome domain now rests on a single study. Against a comparator providing no arch support, one trial showed a large reduction in pain (*g* = −1.017, 95% CI −1.54 to −0.49) on its own designated primary instrument. The same trial's Foot Function Index total gives *g* = −0.28, so the magnitude of that benefit is unstable. Adding short foot exercise to an orthosis worn by both arms reduced pain in one further trial (*g* = −0.974, 95% CI −1.62 to −0.33). That result concerns the exercise rather than the device. One trial showed better dynamic balance at 12 weeks with the orthosis removed (*g* = −0.859, 95% CI −1.59 to −0.13). One within‐subject study showed that orthoses redistribute plantar load away from the heel and lesser metatarsals and toward the medial midfoot. That redistribution is the mechanical action such devices are designed to produce, and it is the most consistent finding in this review. Taken together, the corrected evidence supports the mechanical action of foot orthoses better than it supports any specific clinical benefit. Orthoses remain a reasonable conservative option for flexible flatfoot, but the size of their effect on pain and balance is not established. Adequately powered randomized trials with comparable outcomes and clearly defined comparators are required.

## Author Contributions

**Taeseok Choi:** conceptualization, methodology, data curation, formal analysis, writing – original draft, project administration. **Sangbin Lee:** investigation, resources, visualization, writing – review and editing. **Wansuk Choi:** data curation, investigation, validation, writing – review and editing. **Seoyoon Heo:** data curation, investigation, validation, writing – review and editing.

## Funding

This work was supported by Namseoul University.

## Ethics Statement

Ethical approval was not required for this systematic review as it involved analysis of previously published studies.

## Consent

The authors have nothing to report.

## Conflicts of Interest

The authors declare no conflicts of interest.

## Supporting information


Supporting Information S1

Supporting Information S2

## Data Availability

The data that supports the findings of this study are available in the supplementary material of this article.
