# Supplementary material for: Effects of Orthotic Interventions on Pain, Balance, and Plantar Pressure in Flexible Flatfoot: A Systematic Review
Source: J Foot Ankle Res. 2026 Sep 30;19(4):e70218. doi: 10.1002/jfa2.70218 (PMC13627762; doi:10.1002/jfa2.70218)
Supplement: Supplementary file 1 [file JFA2-19-e70218-s001.docx]

**Supporting Information S2. Characteristics of the eight included studies**

Participant numbers are individuals, not datasets or feet. Where a study measured the same participants under more than one condition, or contributed more than one arm to a single contrast, the participants are counted once. No outcome domain in this review is pooled, and the second table below gives the comparator stratum of every study and states, for each, whether it contributes an estimate and on what ground it does not.

| **Study** | **Design and allocation** | **Participants** | **Population** | **Intervention arms** | **Comparator** | **Co-interventions** | **Follow-up** | **Outcome domains** |
| --- | --- | --- | --- | --- | --- | --- | --- | --- |
| Taspinar 2017 | Nonrandomized controlled trial. Allocation by patient preference: "We could not randomize our three groups" | 60 (20 / 20 / 20) | Adults with bilateral pes planus, outpatient clinic, Turkey | Internal shoe modification (medial arch support insole); external shoe modification (Thomas heel) | Home exercise programme, no orthotic device | Disease education and monthly outpatient review to all three arms. The exercise programme was the comparator itself and was withheld from both device arms | 3 months | Pain (Foot Pain Index, Foot Function Index pain subscale); plantar pressure measured but no group summary statistics published |
| Yurt 2019 | Randomized parallel-group trial. Computer-generated list, opaque envelopes, independent enroller; intention-to-treat | 67 (22 / 22 / 23) | Adults with painful flexible flatfoot, mean age about 22 years, Turkey | CAD-CAM insole; conventionally manufactured insole | Sham flat 3 mm EVA insole | The same home exercise programme was given to all three arms, including the sham arm, so it does not confound the contrast | 8 weeks | Pain (100 mm visual analogue scale) |
| Xu 2019 | Randomized parallel-group trial. Computer-generated randomization by an independent therapist | 80 (40 / 40) | Adults with symptomatic bilateral flexible flatfoot, mean age about 40 years, China | Customised 3D-printed insole | Prefabricated insole (an alternative orthosis, not a no-orthosis control) | None. Standardised flat-based shoes were worn by both arms | 8 weeks | Plantar pressure (in-shoe, ten masked regions) |
| Khodaei 2017 | Nonrandomized within-subject study. Non-random convenience sampling; three conditions in one session | 19 (same participants in all conditions) | Adults with flexible flatfeet, university students, mean age about 23 years, Iran | Prefabricated foot orthosis; CAD-CAM custom foot orthosis | Shoe-only condition | None possible; a single laboratory session with the same standardised sneaker in every condition | None; immediate in-shoe effects only | Plantar pressure (Pedar-X, eight masked regions) |
| Liebau 2023 | Randomized trial with a partial crossover at six months. Allocation by an independent party; double-blind | 52 (21 / 21 / 10) | Children with flexible flatfoot, mean age 8.2 years, Germany | Supportive arch-support insole; sensorimotor insole | Placebo insole, visually identical | The same daily home foot-exercise programme was given to all three arms, including the placebo arm | 6 and 12 months | Pain measured on a numeric rating scale but no numeric data published; plantar contact area measured barefoot |
| Elsayed 2023 | Randomized parallel-group trial. 1:1 permuted-block allocation; assessor-blinded; intention-to-treat with last observation carried forward | 40 (20 / 20) | Adults with symptomatic flexible flatfoot, mean age about 25 years, Saudi Arabia | Prefabricated insole plus short foot exercises | The same prefabricated insole alone, worn 8 hours a day by both arms (adherence 83.2 versus 82.2 per cent, P = 0.83) | Short foot exercises were given to the experimental arm only. This is the only study in which a co-intervention was not balanced across arms | 6 weeks | Pain (numeric pain rating scale); plantar pressure (whole foot, unmasked) |
| Yalfani 2023 | Randomized parallel-group trial. Evaluators blinded; participants could not be blinded, since the comparator arm received no device | 30 (15 / 15) | Adolescent girls, competitive taekwondo athletes with flexible flatfoot, mean age 13.5 years, Iran | Prefabricated foot orthosis with medial arch support | No intervention. The only control arm in the review that received neither a device nor any other treatment | None delivered by the study. Both arms wore the same standardised taekwondo shoe | 12 weeks | Balance and postural stability (Biodex overall stability index) |
| Saeedi 2024 | Randomized within-subject study. Only the order of the four conditions was randomized, by computer-generated random number | 34 (same participants in all conditions) | Adults with bilateral symptomatic flat feet, university orthotic clinic, Iran | Modified UCBL foot orthosis (MUFO); UCBL insole | Shoe-only and barefoot conditions | None. The same standard sport shoe was worn in every shod condition | None; 2-week acclimatisation then single-session testing | Centre-of-pressure displacement during gait |

**Notes**

Total participants across the eight studies: 382. Counting each participant once, the participants who contribute a reported estimate are: pain 67 in stratum A (Yurt) and 40 in stratum C (Elsayed); balance and postural control 30 (Yalfani); plantar loading 19 (Khodaei). The remaining 226 participants are in studies that contribute no estimate, for the reasons given in the second table. Elsayed 2023 also measured plantar pressure, but that contribution is not admitted, so no participant is counted in more than one domain.

Six of the eight studies allocated participants at random. Taspinar 2017 allocated by patient preference and Khodaei 2017 used non-random convenience sampling; because RoB 2 presupposes randomized allocation and does not assess confounding, these two were additionally appraised with ROBINS-I, Taspinar 2017 at critical risk on the confounding domain and Khodaei 2017 at serious risk overall. Saeedi 2024 randomized the order of conditions within participants rather than allocating participants to groups, and Liebau 2023 incorporates a crossover at six months. No single collective label is applied to the eight; each design is named where it bears on the analysis.

Yalfani 2023 provided the only control arm in the review that received neither a device nor any other treatment. The remaining comparators were a sham or placebo insole (Yurt, Liebau), a shoe-only or barefoot condition (Saeedi, Khodaei), an alternative orthosis (Xu), the same orthosis in both arms (Elsayed), or an exercise programme (Taspinar).

**Comparator strata and contribution to the review**

Contrasts were classified by what the comparator provided: stratum A, an orthosis against a comparator providing no arch support, whether a sham insole, a shoe alone, or no device; stratum B, one orthosis against a different orthosis; and stratum C, an orthosis with an added adjunct intervention against the same orthosis worn alone. The three strata estimate different quantities, and an estimate from one stratum was never combined with an estimate from another.

| **Study** | **Comparator stratum** | **Contributes to the review** | **Ground, where it does not contribute** |
| --- | --- | --- | --- |
| Yurt 2019 | A: orthosis versus a comparator providing no arch support (sham flat 3 mm EVA insole) | Pain, stratum A. The two active orthosis arms were combined by the Cochrane Handbook formula so that the shared sham arm of 23 participants is counted once. | - |
| Yalfani 2023 | A: orthosis versus no orthosis | Balance and postural control. Measured barefoot at 12 weeks with the device removed. | - |
| Khodaei 2017 | A: orthosis versus shoe only | Plantar loading, as eight region-specific estimates. Within-subject; the two orthosis conditions were combined per participant, so the 19 participants enter each regional estimate exactly once. | - |
| Elsayed 2023 | C: orthosis with an added adjunct intervention versus the same orthosis alone | Pain, stratum C, reported separately. Both arms wore the identical insole, so the contrast estimates the added short foot exercise and not the device. | Its plantar contribution is not admitted: the reported plantar variables are whole-foot and unmasked, and the contrast is a stratum C contrast in any case. |
| Taspinar 2017 | A: orthosis versus a home exercise programme, no device | Nothing. | The published standard error of 0.215 cannot arise from a 20-versus-20 comparison, whose floor is 0.3162 before and 0.3099 after the Hedges correction. An exhaustive grid of 84 candidate computations reproduces neither published effect size. Allocation was by patient preference with significant baseline imbalance, giving a critical risk of bias on the ROBINS-I confounding domain, and endpoint and change-score analyses point in opposite directions. |
| Liebau 2023 | A: orthosis versus a visually identical placebo insole | Nothing. | The pre-specified pain instrument, a numeric rating scale, is not reported numerically anywhere in the paper. Selecting whichever pain-related value the report does contain would substitute our choice of outcome for the trial’s own. |
| Saeedi 2024 | A: orthosis versus shoe-only and barefoot conditions | Nothing. | Centre-of-pressure was measured with the device in situ in a single session. Yalfani 2023 measured barefoot at 12 weeks with the device removed. The two are opposite measurement conditions and estimate different quantities, so they cannot be pooled. Reported narratively in the Results. |
| Xu 2019 | B: one orthosis versus a different orthosis | Nothing. | The control arm wore a prefabricated foot orthosis, so the contrast is device against device and belongs to a different stratum. The candidate cell was also imbalanced at baseline. |

Applying these rules leaves one estimate in every outcome domain, so no domain is pooled and no summary diamond appears anywhere in this review. Excluding the two studies that did not allocate participants at random changes nothing in the pain and balance domains, where Taspinar 2017 is already withdrawn on arithmetic grounds and the contributing trials randomized, but empties the plantar loading domain entirely, because Khodaei 2017 is the only study admitted to it.
