# Supplementary material for: Effects of Orthotic Interventions on Pain, Balance, and Plantar Pressure in Flexible Flatfoot: A Systematic Review
Source: J Foot Ankle Res. 2026 Sep 30;19(4):e70218. doi: 10.1002/jfa2.70218 (PMC13627762; doi:10.1002/jfa2.70218)
Supplement: Supplementary file 2 [file JFA2-19-e70218-s002.docx]

**Supporting Information S1. Database search strategies**

APPENDIX: COMPLETE SEARCH STRATEGIES FOR REPRODUCIBILITY

--------------------------------------------------------

Search Date: October 16, 2025

Language Restrictions: English only (full-text availability required)

Database Inception: Inception to October 16, 2025

Grey Literature: NOT searched (peer-reviewed studies only)

Clinical Trial Registries (ClinicalTrials.gov, ISRCTN): NOT searched

**1. PUBMED (MEDLINE) SEARCH STRATEGY**

-----------------------------------

**Search Terms (Combined):**

("Foot Orthoses" OR "Orthotic Devices" OR "Foot Arch Supports" OR "Orthotic Shoe Inserts" OR "foot orthosis" OR "shoe inserts" OR "arch supports")

**AND**

("Flatfoot" OR "Pes Planus" OR "Flexible Flatfoot" OR "pes planus" OR "flat foot" OR "collapsed arch")

**MeSH Terms Used:**

- Foot Orthoses [MeSH] (exploded)

- Flatfoot [MeSH] (exploded)

- Orthotic Devices [MeSH]

- Foot [MeSH]

Results: 178 articles

**Additional Filters Applied:**

- Study Design: Randomized Controlled Trials, Clinical Trials (optional filter applied)

- Date: Inception to October 16, 2025

- Language: English

**2. EMBASE SEARCH STRATEGY (ELSEVIER)**

------------------------------------

**Search Terms (Adapted for EMTREE):**

('foot orthosis':ti,ab,kw OR 'orthotic device':ti,ab,kw OR 'foot arch support':ti,ab,kw OR 'shoe insert':ti,ab,kw OR 'arch support':ti,ab,kw)

**AND**

('flatfoot':ti,ab,kw OR 'pes planus':ti,ab,kw OR 'flexible flatfoot':ti,ab,kw OR 'flat foot':ti,ab,kw OR 'collapsed arch':ti,ab,kw)

**EMTREE Controlled Terms:**

- Foot Orthosis (preferred term)

- Orthotic Device

- Flatfoot

- Foot Arch

Results: 40 articles

**Additional Filters Applied:**

- Study Type: Randomized Controlled Trial, Clinical Trial (optional)

- Date: Inception to October 16, 2025

- Language: English

**3. COCHRANE LIBRARY SEARCH STRATEGY**

-----------------------------------

**Search Terms (Adapted for Cochrane):**

("Foot Orthoses" OR "Orthotic Devices" OR "Foot Arch Supports" OR "Orthotic Shoe Inserts")

**AND**

("Flatfoot" OR "Pes Planus" OR "Flexible Flatfoot")

Cochrane-Specific Fields:

- Title, Abstract, Keywords

Results: 72 articles

**Search Filters Applied:**

- Database: Cochrane Central Register of Controlled Trials (CENTRAL)

- Study Type: Randomized Controlled Trials

- Date: Inception to October 16, 2025

- Language: English

**SUMMARY OF SEARCH RESULTS**

-------------------------

| **Database** | **Articles found** | **Duplicates** | **After deduplication** |
| --- | --- | --- | --- |
| PubMed (MEDLINE) | 178 | — | — |
| EMBASE | 40 | — | — |
| Cochrane Library | 72 | — | — |
| **Total** | **290** | **−45** | **245** |

Duplicates were identified across databases and could not be attributed to any single source; the count is therefore given only for the total.

Final Included Studies: 8 studies

**RATIONALE FOR SEARCH STRATEGY**

-----------------------------

1. Database Selection:

- PubMed (MEDLINE): Primary biomedical database; most comprehensive for

orthopedic and rehabilitation studies.

- EMBASE (Elsevier): Complements PubMed; includes European and Asian

journals not indexed in MEDLINE.

- Cochrane Library: Specialized systematic review resource; indexes

high-quality RCTs.

2. Search Term Rationale:

- "Foot Orthoses" and synonyms: Captures various orthotic interventions

(custom, prefabricated, arch supports, insoles).

- "Flatfoot" and synonyms: Ensures inclusion of all terminology for

flexible flatfoot (pes planus, collapsed arch).

- AND Boolean operator: Ensures both intervention AND condition are present.

3. Language Restriction:

- English only: Reduces bias from language translation; ensures feasibility

of data extraction and risk-of-bias assessment by the review team.

- Implication: Potential language bias; studies published in other languages

(especially Asian journals) may be missed.

4. Exclusions:

- No grey literature search (dissertations, conference abstracts, preprints).

- No clinical trial registry search.

- Rationale: Focus on peer-reviewed evidence with established validity;

reduces risk of publication bias from unpublished trials.

**UPDATES AND MODIFICATIONS**

-------------------------

Date of Last Update: October 16, 2025

Future Updates: Systematic review completed; no planned updates.

Modifications from Original Protocol: The search was run to 16 October 2025 rather than to the 16 February 2025 date registered; the protocol provided for the search to be updated before the final analysis, so this is an update rather than a departure. Screening of the reference lists of included reports was specified in the protocol and was not carried out. Both are recorded in the amendment to PROSPERO CRD42025649490, together with the other deviations declared in the manuscript. The search strategy itself remained consistent with PROSPERO registration (CRD42025649490).
